# Factor H Binds to the Hypervariable Region of Many *Streptococcus pyogenes* M Proteins but Does Not Promote Phagocytosis Resistance or Acute Virulence

**DOI:** 10.1371/journal.ppat.1003323

**Published:** 2013-04-18

**Authors:** Mattias C. U. Gustafsson, Jonas Lannergård, O. Rickard Nilsson, Bodil M. Kristensen, John E. Olsen, Claire L. Harris, Rafael L. Ufret-Vincenty, Margaretha Stålhammar-Carlemalm, Gunnar Lindahl

**Affiliations:** 1 Medical Microbiology Section, Department of Laboratory Medicine, Medical Faculty, Lund University, Lund, Sweden; 2 Department of Veterinary Disease Biology, Faculty of Health and Medical Sciences, University of Copenhagen, Copenhagen, Denmark; 3 Institute of Infection and Immunity, School of Medicine, Cardiff University, Cardiff, United Kingdom; 4 Department of Ophthalmology, University of Texas Southwestern Medical Center, Dallas, Texas, United States of America; New York Medical College, United States of America

## Abstract

Many pathogens express a surface protein that binds the human complement regulator factor H (FH), as first described for *Streptococcus pyogenes* and the antiphagocytic M6 protein. It is commonly assumed that FH recruited to an M protein enhances virulence by protecting the bacteria against complement deposition and phagocytosis, but the role of FH-binding in *S. pyogenes* pathogenesis has remained unclear and controversial. Here, we studied seven purified M proteins for ability to bind FH and found that FH binds to the M5, M6 and M18 proteins but not the M1, M3, M4 and M22 proteins. Extensive immunochemical analysis indicated that FH binds solely to the hypervariable region (HVR) of an M protein, suggesting that selection has favored the ability of certain HVRs to bind FH. These FH-binding HVRs could be studied as isolated polypeptides that retain ability to bind FH, implying that an FH-binding HVR represents a distinct ligand-binding domain. The isolated HVRs specifically interacted with FH among all human serum proteins, interacted with the same region in FH and showed species specificity, but exhibited little or no antigenic cross-reactivity. Although these findings suggested that FH recruited to an M protein promotes virulence, studies in transgenic mice did not demonstrate a role for bound FH during acute infection. Moreover, phagocytosis tests indicated that ability to bind FH is neither sufficient nor necessary for *S. pyogenes* to resist killing in whole human blood. While these data shed new light on the HVR of M proteins, they suggest that FH-binding may affect *S. pyogenes* virulence by mechanisms not assessed in currently used model systems.

## Author Summary

The human complement system may be rapidly activated upon infection and thereby plays a key role in innate immunity. However, activation must be tightly controlled, to avoid attack on self tissues. A key component of this control system is the plasma protein factor H (FH). Many pathogens bind FH, as first described for *Streptococcus pyogenes*, and it has been proposed that the surface-localized M protein of this bacterium “hijacks” FH to escape phagocytosis. However, it remains unclear whether FH-binding to M protein indeed protects *S. pyogenes* against phagocytosis and promotes bacterial growth *in vivo*. Here, we demonstrate that FH binds to some but not all M proteins and solely binds to the hypervariable region (HVR), a part of M protein important for virulence. Nevertheless, several lines of evidence, including studies with transgenic mice, indicated that FH-binding ability did not contribute to acute virulence or phagocytosis resistance. These data shed new light on the HVR of M proteins but underline the difficulty in determining the *in vivo* role of a ligand-binding region. Binding of FH may contribute to *S. pyogenes* virulence by mechanisms not assessed in currently used models.

## Introduction

The human complement system plays a key role in the defense against infections, in inflammatory reactions, and in immune responses [1], [2]. Fulfillment of these roles requires complement activation, which may proceed via either of three pathways, the classical, lectin and alternative pathways. The alternative pathway plays a particularly important role in promoting innate immunity to infections, because it is continuously activated at a low level and includes an efficient amplification loop, allowing rapid activation and attack on an infecting pathogen [3]. Accordingly, the alternative pathway must be tightly controlled to avoid excess complement activation. A major component of this control system is the ∼150 kDa protein factor H (FH), which is present both free in plasma and bound to cell surfaces, where it down-regulates complement activation [4], [5]. Lack of FH causes uncontrolled activation via the alternative pathway and kidney disease, demonstrating the *in vivo* importance of this regulator [6].

FH not only binds to host cell surfaces but also binds to surface proteins of many pathogenic bacteria, as first reported for the M6 protein of *Streptococcus pyogenes* (group A streptococcus) [7]. In the currently favored model, FH is recruited to M protein to protect the bacteria from complement attack and rapid killing, in particular through phagocytosis [8]–[10]. It may seem intuitively obvious that this model must be correct, possibly explaining why it is presented as a fact in numerous publications and review articles and even in textbooks [11], [12]. However, to our knowledge there is no conclusive evidence that FH bound to M protein promotes virulence, i.e. growth *in vivo*. This situation prompted us to study the interaction between FH and *S. pyogenes* M protein, with focus on the possible role of bacteria-bound FH in virulence.

*S. pyogenes* is a Gram-positive bacterium that causes a variety of diseases, including superficial throat and skin infections, streptococcal toxic shock syndrome, and the autoimmune disease rheumatic fever [13]. The fibrillar M protein, which is the most extensively studied virulence factor of *S. pyogenes*, is a surface-anchored coiled-coil protein that prevents phagocytosis [14]. A characteristic feature of this protein is the presence of an N-terminal hypervariable region (HVR), which has a length of ∼50–100 amino acid residues and is a key target for type-specific protective antibodies [14]. The HVR exhibits extreme sequence divergence among M proteins expressed by different strains but is typically stable within a strain, allowing the identification of ∼200 distinct M (*emm*) types [15].

Although the ability of M protein to bind FH has attracted much interest, the location of the binding site for FH has remained unclear. An early study reported that FH binds to the conserved C repeat region of the M6 protein, implying that all M proteins may bind FH via this conserved region [16]. However, another study indicated that FHL-1, a naturally occurring minor FH splice variant that would be expected to bind to the same site as FH, interacted with the HVR of the M5 and M6 proteins, and did not bind to the M22 protein [17]. Combining these two results, one report suggested that FH and FHL-1 bind to both the HVR and the C-repeat region of an M protein [18]. Thus, it is unclear whether all M proteins bind FH and where FH binds in an M protein.

The biological role of FH/FHL-1 bound to M protein has similarly remained unclear [18]–[20]. Analysis with pure proteins indicated that bacteria-bound FH/FHL-1 retains its complement regulatory activity, suggesting that recruitment of the human protein indeed protects *S. pyogenes* against complement attack and phagocytosis [7], [17]. However, studies of bacteria suspended in human plasma suggested that, at least for FH, binding is largely blocked by fibrinogen (Fg), implying that FH may bind poorly to M protein under physiological conditions [17], [21]. In agreement with this finding, the ability of an M protein to bind FH/FHL-1 had little effect on complement deposition, when the analysis was performed in human plasma [18]. The latter study also suggested that binding to the HVR is of limited importance for phagocytosis resistance, but since the authors reported that FH not only binds to the HVR, but also binds to the C repeats, the results did not exclude that binding to the C repeats was sufficient to promote phagocytosis resistance. Thus, the role of FH-binding remains controversial, as witnessed by two recent reports, which suggested that FH-binding indeed promotes phagocytosis resistance [22], [23].

Here, we studied the ability of different M proteins to bind FH, the site of FH-binding in an M protein, and the biological role of the binding. Using seven highly purified M proteins, we found that human FH binds to some (but not all) M proteins and binds solely to the HVR, which represents a distinct FH-binding domain. Unexpectedly, studies in a transgenic mouse model did not support the hypothesis that bound FH promotes virulence during the acute stage of an infection. Moreover, assays in a human whole blood system indicated that FH-binding is neither sufficient nor necessary for phagocytosis resistance.

## Results

### Proteins studied

**Figure 1:**
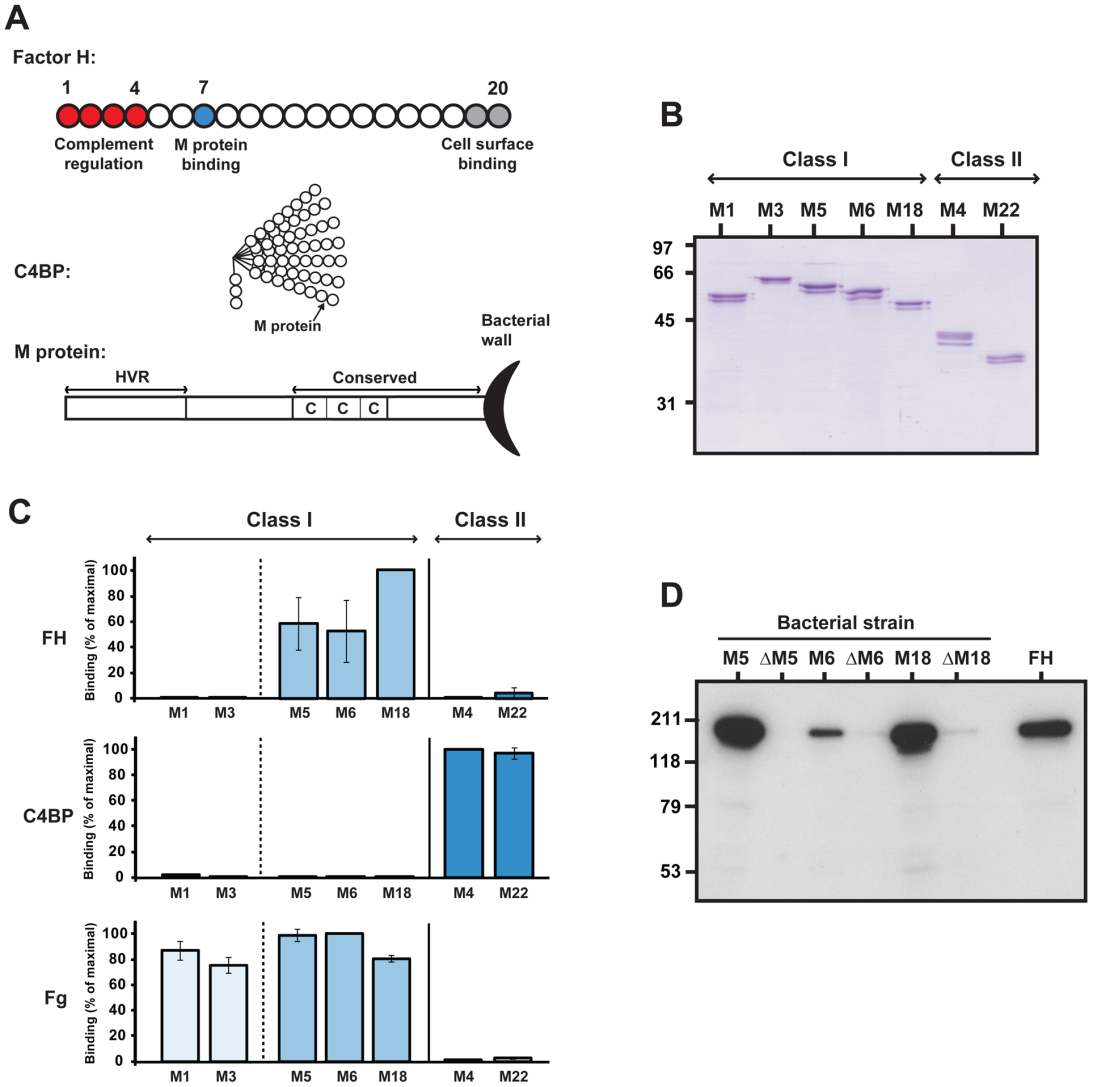
Streptococcal M proteins vary in ability to bind human FH and C4BP. (**A**) Schematic representation of the human complement regulators FH and C4BP and of an M protein. For FH and C4BP, each circle represents an SCR domain. While FH is a single chain with 20 SCR domains, C4BP typically contains 7 α-chains with 8 SCR domains and one β-chain with 3 SCRs. An M protein has an N-terminal HVR and a more conserved C-terminal region that includes C repeats and the wall-anchoring region. The location of M protein binding sites in FH and C4BP are indicated. (**B**) SDS-PAGE analysis of the purified recombinant M proteins studied, five of which were of class I, and two of class II. The presence of doublet or triplet bands is typical for M proteins expressed in *E. coli* or *S. pyogenes* [32], [82]. (**C**) Binding of human FH, C4BP or fibrinogen (Fg) to pure M proteins immobilized in microtiter wells. The wells were coated with 0.1 µg M protein and human ligands (50 µl) were added at the following concentrations: FH 2 µg/ml, C4BP 1 µg/ml, Fg 0.28 µg/ml. Bound ligands were detected with specific antibodies. Binding is given in percent of maximal binding for each ligand. (**D**) Binding of human FH analyzed for the M-positive M5, M6 and M18 *S. pyogenes* strains and their M-negative mutants (ΔM5, ΔM6, and ΔM18, respectively). Bacterial suspensions were incubated with pure FH (50 µg/ml). After two washes bound protein was eluted and analyzed by western blot, employing anti-FH for detection. Pure FH was included as a control in the blot (right).

The proteins studied here are shown schematically in Figure 1A. The FH molecule is composed of 20 short consensus repeat (SCR) domains, of which SCR1-4 are essential for complement regulatory activity, while SCR19-20 promote binding to polyanions on human cell surfaces [4], [5]. A site in SCR7 has been implicated in the binding of M proteins. The presence of histidine (H) rather than tyrosine (Y) at position 402 in this site reduces the affinity between FH and the M6 protein [24] and increases the risk for the common eye disease age-related macular degeneration (AMD) [25].

Some tests were performed with C4BP, which like FH is a major complement regulator present in human plasma. This ∼570 kDa protein down-regulates the classical and lectin pathways, is a member of the same protein family as FH, and binds to many M proteins, which have a C4BP-binding site in the HVR [26]–[28].

The seven highly purified recombinant M proteins studied here were of either class I or class II, the two major classes of M proteins [29], and had the expected molecular mass, as demonstrated by SDS-PAGE (Figure 1B). Moreover, they had the expected N-terminal sequence, as shown by Edman degradation, demonstrating that their HVRs were intact (data not shown). The class I proteins included M1 and M3, two of the most common M types associated with invasive infections in the western world [30], as well as M5, M6 and M18, which have been epidemiologically associated with rheumatic fever [29]. Thus, the five class I proteins were of serotypes associated with the two most important life-threatening diseases caused by *S. pyogenes* [13]. The two class II proteins, M4 and M22, are clinically common and have been extensively studied [31]–[33].

### M proteins vary in ability to bind FH or C4BP

Although this study was focused on FH, it was of interest to compare several M proteins for ability to bind the two structurally related complement regulators FH and C4BP. For this purpose, the seven purified M proteins were immobilized in microtiter wells and tested for binding of the two human proteins (Figure 1C). Thus, the analysis was performed under non-denaturing conditions. As a control, the M proteins were analyzed for ability to bind fibrinogen (Fg), a ligand that binds to all class I proteins but not to class II proteins, according to current knowledge [34]. In this analysis, the class I proteins M1 and M3 did not bind FH or C4BP but showed binding of Fg, as expected. In contrast, binding of FH was observed for the three class I proteins M5, M6 and M18. The two class II proteins showed good binding of C4BP but little or no binding of FH. For clarity of presentation, the data in Figure 1C represent results obtained with a single concentration of human ligand, but the binding was concentration dependent, as shown for FH in Figure S1. These binding data with FH and C4BP extend and confirm previous studies [7], [17], [18], [26], [27], [35] and indicate that ability to bind FH is a property of some but not all M proteins.

Our data indicated that several M proteins are unable to bind FH, but did not formally exclude that these M proteins bind FH with such a low affinity that binding was not detected with the standard methods used. However, striking differences clearly exist among the M proteins studied here. Indeed, our data suggest that M proteins may be divided into at least three groups, representing proteins that selectively bind FH, selectively bind C4BP, or bind neither of these human ligands.

Subsequent analysis was focused on the three FH-binding M proteins, M5, M6 and M18. To analyze whether M protein is the only FH-binding surface protein of the corresponding strains, we incubated wild type bacteria and isogenic M-negative mutants with FH (Figure 1D). After incubation and washes, bound FH was eluted from the bacteria. While the M-positive strains showed good binding of FH, little or no binding was observed for the M-negative mutants, indicating that the M-positive strains express one major FH-binding protein, the M protein. It is of particular interest that the M5-negative strain was completely unable to bind FH because this result indicates that the M5 strain, which was used for infection experiments described later, expresses a single FH-binding protein, the M5 protein. Of note, this analysis with whole bacteria was performed with a relatively high concentration of FH (50 µg/ml) and few washes, suggesting that a low-affinity binding to a surface structure different from M5 might have been detected, but no binding was seen.

### The FH-binding site is located in the HVR of an M protein

**Figure 2:**
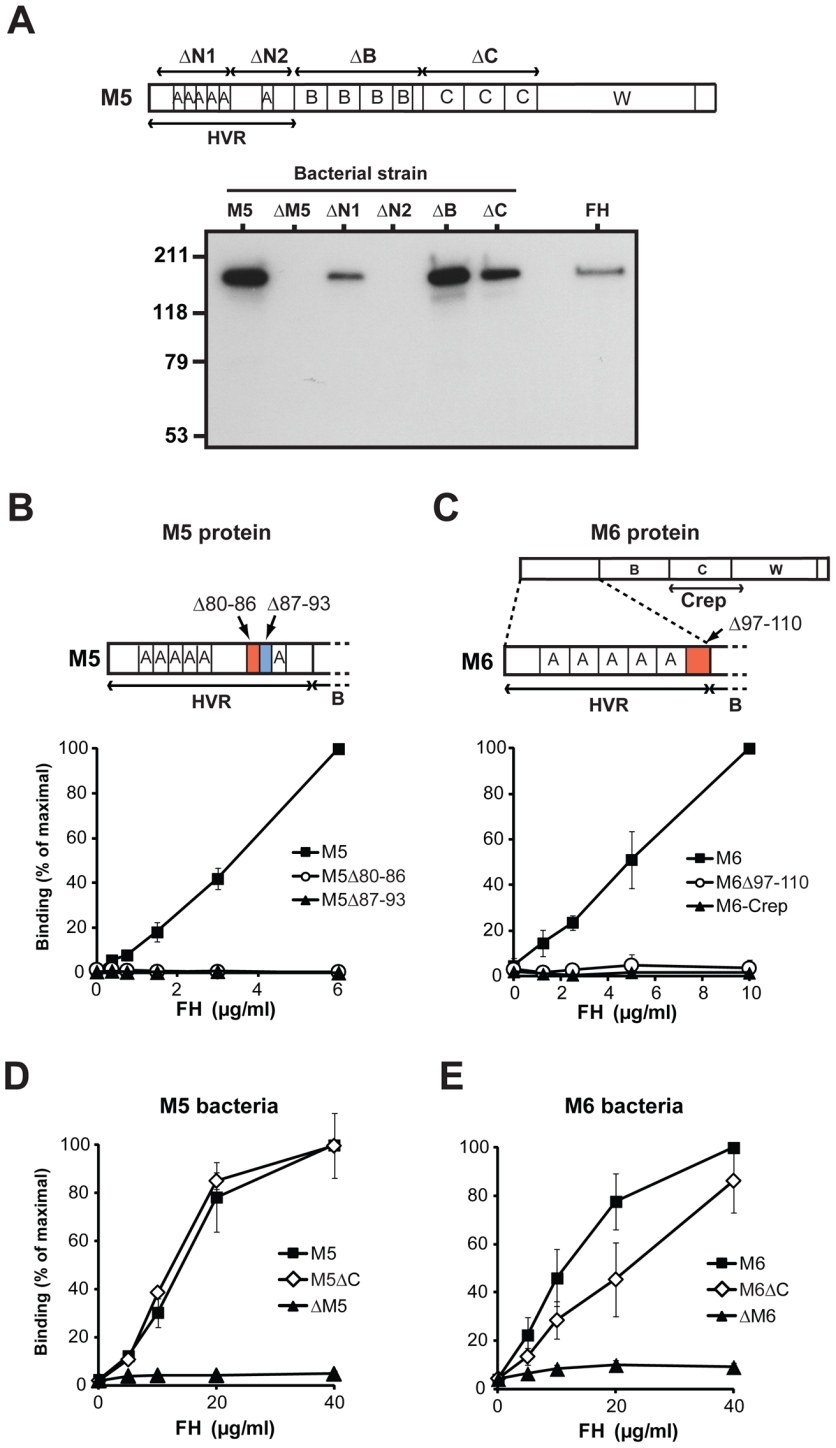
Human FH binds to the HVR of the M5 and M6 proteins. (**A**) Binding of human FH to chromosomal mutants of the M5 strain. The upper part of the panel shows the location of deletions (ΔN1, ΔN2, ΔB and ΔC) causing the surface expression of truncated M5 proteins, and the positions of A, B and C repeats in M5. The lower part of the panel shows results of binding tests with pure FH and the mutants expressing truncated M5 proteins. The wild type M5 strain and a strain (ΔM5) lacking the entire M5 protein were also included. In the binding tests, pure FH was incubated with a suspension of the strain indicated. After washes, bound protein was eluted from the bacteria and analyzed by western blot, employing anti-FH for detection. Pure FH was included as a control in the blot (right). (**B** and **C**) Binding of FH to purified derivatives of the M5 protein (B) and the M6 protein (C). The proteins indicated were used to coat microtiter wells, using 0.1 µg per well, and analyzed for ability to bind pure FH, added at the concentration indicated. In B, wild type M5 protein was compared with two M5 derivatives (M5Δ80-86 and M5Δ87-93) with short deletions in the HVR, as indicated. In C, wild type M6 protein was compared with a deletion derivative having a short deletion in the HVR (M6Δ97-110), and with a dimerized construct derived from the C repeat region of M6 (M6-Crep), as indicated. (**D** and **E**) Binding of FH to whole *S. pyogenes* M5 bacteria (D) or M6 bacteria (E), and to bacterial mutants of these strains lacking C repeats (M5ΔC and M6ΔC) or no M protein (ΔM5 and ΔM6).

To identify the binding site(s) for FH in an M protein, we first studied the M5 protein, employing a series of isogenic chromosomal deletion mutants of the *S. pyogenes* M5 strain (Figure 2A). These mutant strains lacked the entire M5 protein (strain ΔM5) or expressed truncated M5 proteins lacking parts of the protein (strains ΔN1, ΔN2, ΔB and ΔC). The truncated M5 proteins and the wild-type protein are expressed at similar levels on the surface, allowing direct comparisons of the corresponding strains [20]. Incubation of bacterial suspensions with FH showed binding to all truncated mutant proteins except ΔN2, which lacks the C-terminal half of the HVR. The simplest explanation for these data is that FH binds to a single site, located in the N2 region of the M5-HVR.

To study the FH-binding site in M5 under different conditions, we constructed two M5 proteins with a short deletion in the N2 region, the M5Δ80-86 and M5Δ87-93 proteins (Figure 2B and S2A). Each of these two proteins lacked one coiled-coil heptad (7 amino acids). Pure preparations of the deletion proteins and of the intact M5 protein were immobilized in microtiter wells and analyzed for ability to bind FH. In this analysis, the two deletion proteins did not bind FH, supporting the conclusion that FH binds solely to the N2 region of M5 (Figure 2B). Because it has been reported that FH binds to the C repeat region of the M6 protein [16], studies were also performed in that system (Figure 2C and S2A). We hypothesized that FH binds to the C-terminal part of the HVR also in M6, and studied an M6 deletion protein lacking a short sequence (14 amino acids, two heptads) in this part of the HVR. This deletion protein, designated M6Δ97-110, was completely unable to bind FH, suggesting that M6 has a single FH-binding site located in the HVR (Figure 2C). Of note, the lack of FH-binding to the M5 and M6 deletion proteins did not reflect a general inability of these mutant proteins to bind a ligand, because they retained ability to bind fibrinogen, which binds to the B repeats of these M proteins (Figure S2B). Thus, the studies of M5 and M6 deletion proteins supported the conclusion that FH-binding M proteins have a single binding site located in the HVR, while FH does not bind to the C repeat region, which was intact in the deletion proteins.

Given the early report that FH binds to the C repeat region of M6, additional analysis was performed with that M protein. For this purpose, we employed a construct designated M6-Crep, which included the C repeats and was of the same length as an M6 fragment proposed to bind FH [16] (Figure 2C). To promote coiled-coil formation, which may be essential for ligand-binding ability [26], [36], [37], the M6-Crep construct was dimerized via a C-terminal cysteine residue. When analyzed by SDS-PAGE under reducing and non-reducing conditions, this construct was pure and migrated as expected (Figure S2C). After immobilization in microtiter wells, M6-Crep was completely unable to bind FH, like the M6Δ97-110 deletion protein, while good binding was seen for the M6 control (Figure 2C). These data provide further evidence that M6 has a single FH-binding site located in the HVR.

The early immunochemical study, which suggested that FH binds to the C repeats of the M6 protein [16], was supported by studies of whole bacteria. In that analysis, a bacterial M6 mutant lacking C repeats showed reduced binding of FH [19], [38]. Because this result appeared to be at odds with our data, we reanalyzed the role of the C repeats in FH-binding, using *S. pyogenes* bacterial mutants expressing an M5 or M6 protein lacking C repeats. Of note, each of these C repeat mutant strains produces an amount of M protein comparable to that of the corresponding wild type strain, allowing comparison of binding properties [19], [20]. When the wild type strains and the deletion mutants were compared for ability to bind FH, the lack of C repeats had no effect in the M5 system but caused a limited reduction of FH binding in the M6 system (Figure 2D and E). However, the effect in the M6 system was smaller than previously reported [19], [38]. We conclude that the C repeat region is dispensable for FH-binding to whole M5 and M6 bacteria. The limited effect observed in the M6 system might reflect that the HVR probably is located closer to the bacterial cell wall in a mutant lacking C repeats, resulting in steric hindrance. Such a steric effect would not necessarily be seen in the M5 system, because the M5 and M6 strains have cell envelopes of different composition.

### Isolated HVRs derived from M5, M6 or M18 specifically bind FH

To analyze whether the HVR of an FH-binding M protein is sufficient for binding, we tested purified recombinant HVRs. These HVRs were dimerized by means of a C-terminal cysteine residue not present in the intact M protein. As indicated above, previous work had indicated that not only intact M proteins [36], but also domains derived from M proteins, must be present in dimeric form to bind ligands [26], [37], [39]. An isolated domain that is not artificially dimerized via a cysteine residue may be too short to promote formation of a dimeric coiled-coil, unlike the intact M protein.

**Figure 3:**
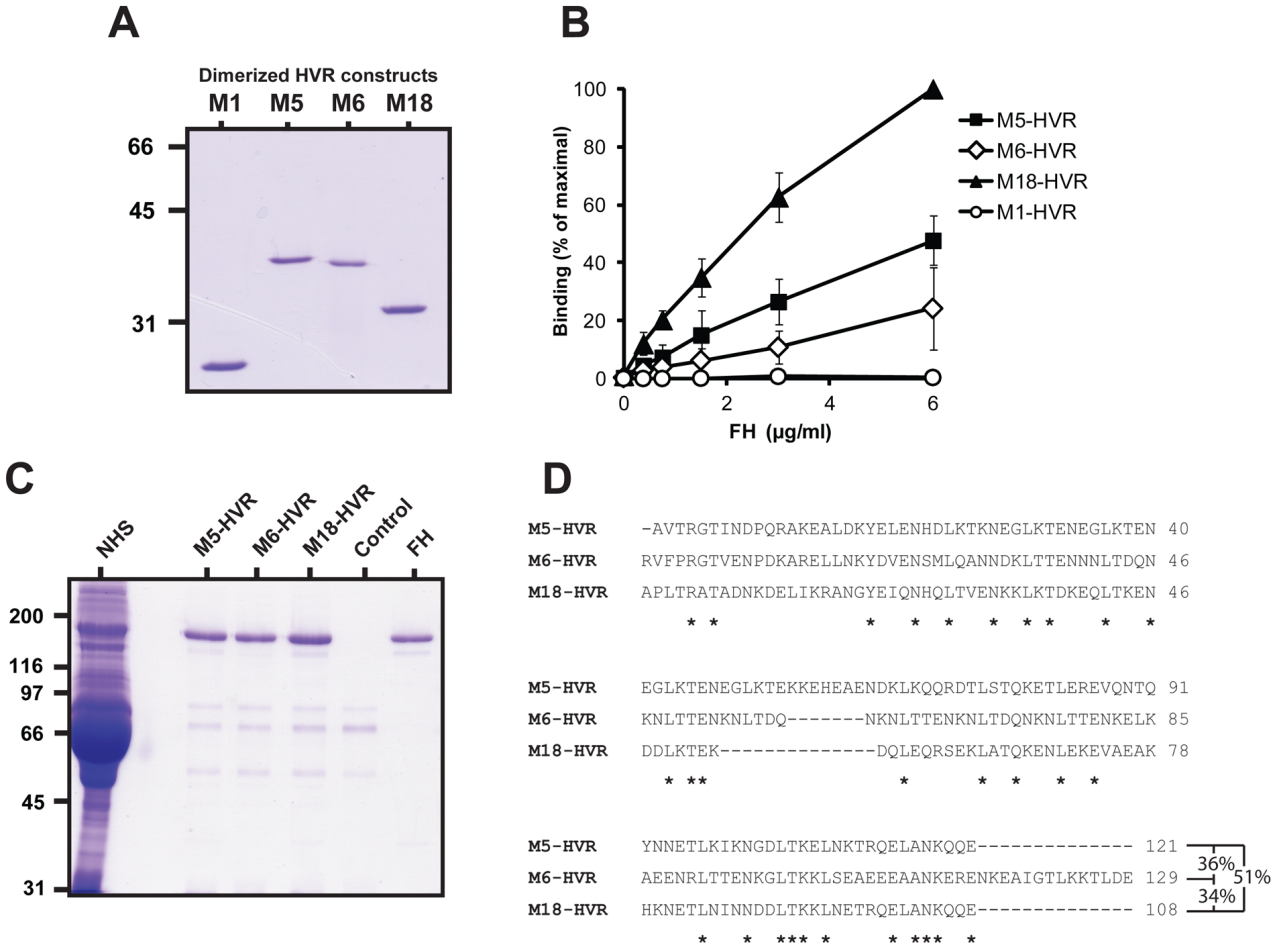
Isolated HVRs derived from M5, M6 and M18 specifically bind FH. (**A**) Analysis by non-reducing SDS-PAGE of HVRs dimerized via a C-terminal cysteine residue. As previously observed [26], dimerized HVRs move more slowly than expected in gels. (**B**) Binding of FH to isolated HVRs immobilized in microtiter wells. The wells were coated with 0.1 µg of the HVRs, as indicated, and tested for ability to bind added FH. (**C**) Immobilized HVRs, derived from FH-binding M proteins, specifically bind FH among all proteins in human serum. Whole human serum was applied to columns in which the HVRs indicated had been immobilized. After washings, bound protein was eluted and analyzed by SDS-PAGE. A column without HVR was used as control. Pure FH was included as a reference in the gel analysis (right). (**D**) Sequence alignment of the three FH-binding HVRs that were studied in isolated form. This alignment does not include a C-terminal Cys residue included to allow dimerization. The lengths of these HVR vary slightly from those previously reported [79], because the position of the C-terminal end was chosen to allow optimal dimerization and FH-binding. Asterisks indicate residues identical in all three sequences. Pair-wise identities (based on regions present in both sequences) are indicated to the right.

Dimerized HVRs were prepared for the FH-binding M5, M6 and M18 proteins and the non-binding M1 protein (Figure 3A). When these HVRs were immobilized in microtiter wells and analyzed for ability to bind FH, binding was observed for the HVRs derived from the FH-binding M proteins, but not for that derived from M1 (Figure 3B). In this test, binding was strongest for the M18-HVR and weakest for the M6-HVR, but all three HVRs derived from FH-binding M proteins could bind FH, in contrast to the M1-HVR, which was completely negative. Thus, isolated HVRs derived from FH-binding proteins retained ability to bind FH.

To analyze the specificity with which the free HVRs interacted with FH, we employed columns containing immobilized HVRs. Whole human serum was passed through such columns, or through a control column without HVR, and bound proteins were eluted. For each of the three FH-binding HVRs, a single protein species dominated in the eluate, as demonstrated by SDS-PAGE (Figure 3C), and this protein was identified as FH by mass spectrometry and western blot (data not shown). No FH was eluted from the control column, but weak SDS-PAGE bands corresponding to polypeptides of lower molecular mass were observed for all eluates, implying that they represented proteins binding to the column matrix. Of note, the eluates from the HVR columns did not contain detectable amounts of the ∼42 kDa FHL-1 protein, which occurs in low concentration in serum and binds to the HVR of the intact M5 protein [17]. Possibly, the HVRs selectively bind FH but not FHL-1 under these conditions. These data show that the three HVRs studied here all bind FH with high specificity, although their sequences show extensive sequence divergence, as seen in an alignment (Fig. 3D). Indeed, no sequence longer than three amino acid residues is shared by all three HVRs.

### FH-binding HVRs show species specificity and bind to the same region in human FH

**Figure 4:**
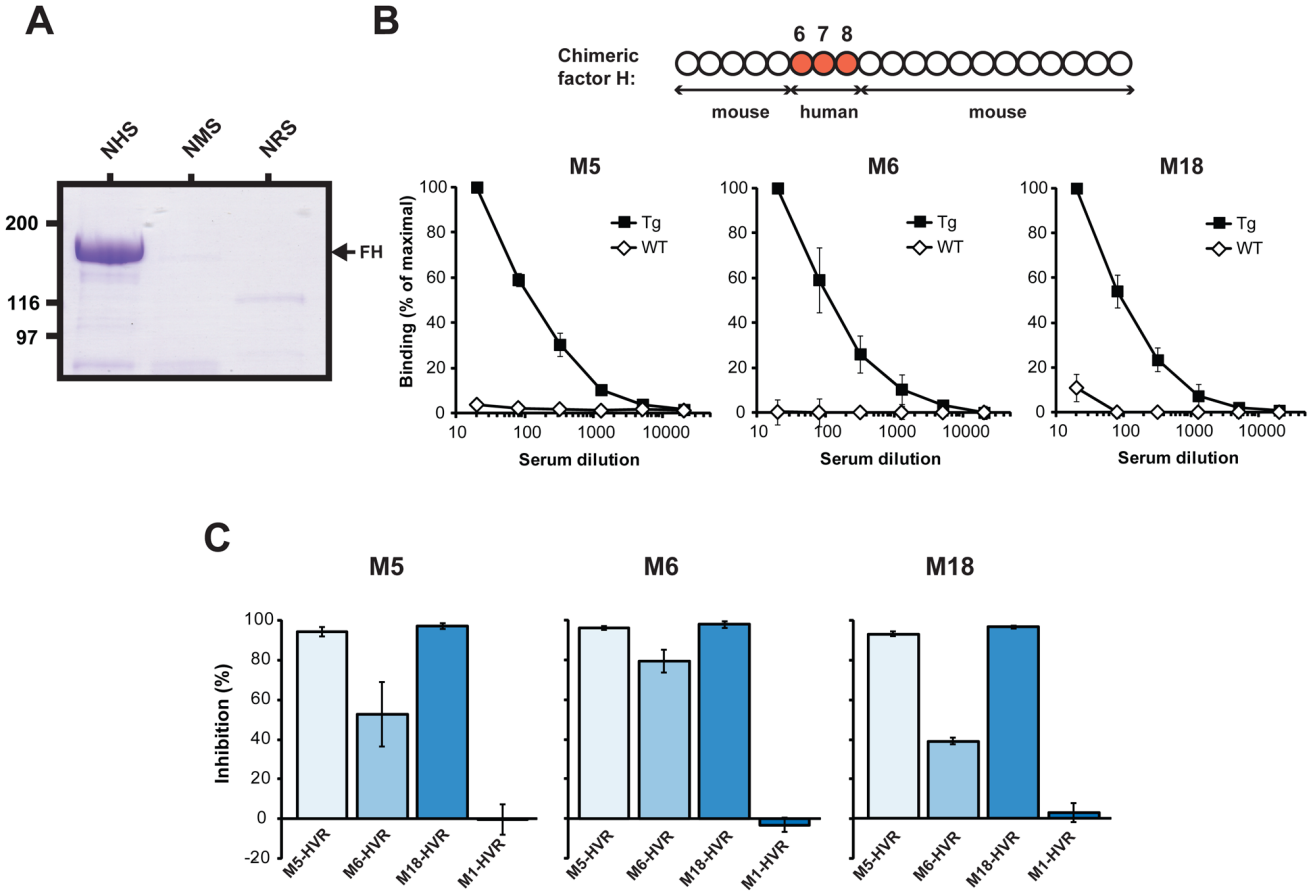
FH-binding M proteins show species specificity and bind to the same region in FH. (**A**) The M5-HVR binds FH present in normal human serum (NHS) but not FH in normal mouse serum (NMS) or normal rabbit serum (NRS). In three separate tests, the sera were applied to a column containing the M5-HVR. After washes, bound protein was eluted and analyzed by SDS-PAGE. (**B**) Binding of a chimeric FH, expressed by Tg mice, to M proteins. The chimeric FH includes the SCR6-8 region of human FH (top). Serum from the Tg mice, or from wild type (wt) C57Bl/6 mice, was analyzed for presence of FH able to bind to the M protein indicated, immobilized in microtiter wells. (**C**) Different FH-binding HVRs bind to the same or overlapping site(s) in FH. A biotinylated form of the M5, M6 or M18 protein was used to detect FH immobilized in microtiter wells, and binding was inhibited with the four HVRs indicated, added at a concentration of 10 µM.

Pathogens commonly show species specificity in their ability to bind a complement regulator, as demonstrated for both C4BP [40]–[42] and FH [43]–[45]. To analyze whether the FH-binding HVRs studied here show species specificity, we applied human, mouse or rabbit serum to columns containing immobilized HVRs and performed the same type of analysis as described in Figure 3C. Binding was only observed for human FH, not for mouse or rabbit FH, as shown for the M5-HVR in Figure 4A. Similar results were obtained for columns containing the M6-HVR or the M18-HVR (data not shown). The lack of recovery of mouse FH was not due to lack of FH in mouse serum, for which the serum concentration of FH is similar to that in humans [6], [46]. For rabbit serum, the concentration of FH is ∼3-fold lower than in human serum [47], but that difference cannot explain the complete lack of FH-binding in our analysis. Thus, the HVRs studied here not only show specificity for FH among all proteins in human serum but also show species specificity.

The available evidence indicates that an M protein binds to a site in SCR7 of human FH [24] (Figure 1A). To confirm that the M proteins studied here have HVRs that bind in this region, we performed two types of analysis. First, we employed serum from transgenic (Tg) mice expressing a chimeric FH, in which only SCRs 6–8 are derived from human FH (Figure 4B, top) [48]. The concentration of chimeric FH in these Tg mice is similar to that of FH in wild-type (wt) mice [48]. The FH present in Tg serum was compared with FH in wt serum for ability to bind to the M5, M6 or M18 proteins, which were immobilized in microtiter wells. Bound FH was detected with a monoclonal antibody directed against the SCR1-4 region of mouse FH. In this analysis, the chimeric FH showed binding to all three M proteins, while FH present in wt mouse serum did not bind (Figure 4B). This result confirms the species specificity of the binding and is in agreement with the reports that M proteins bind to a site in SCR7. Because this analysis employed intact M proteins, the results also indicate that mouse FH does not bind to a site in M proteins located outside of the HVR.

In a second type of analysis, we tested whether the binding of one HVR is inhibited by the other HVRs. For this purpose, the three free HVRs were analyzed for ability to inhibit the binding of an intact M protein to immobilized FH (Figure 4C). The results show that binding of each M protein could be inhibited by the homologous HVR and also by the other two HVRs. In contrast, no inhibition was observed with the HVR derived from the non-FH-binding M1 protein. These data indicate that the HVRs studied here, which have highly divergent sequences, bind to the same or overlapping site(s), most likely in SCR7 of FH.

### Antigenic properties of FH-binding HVRs

**Figure 5:**
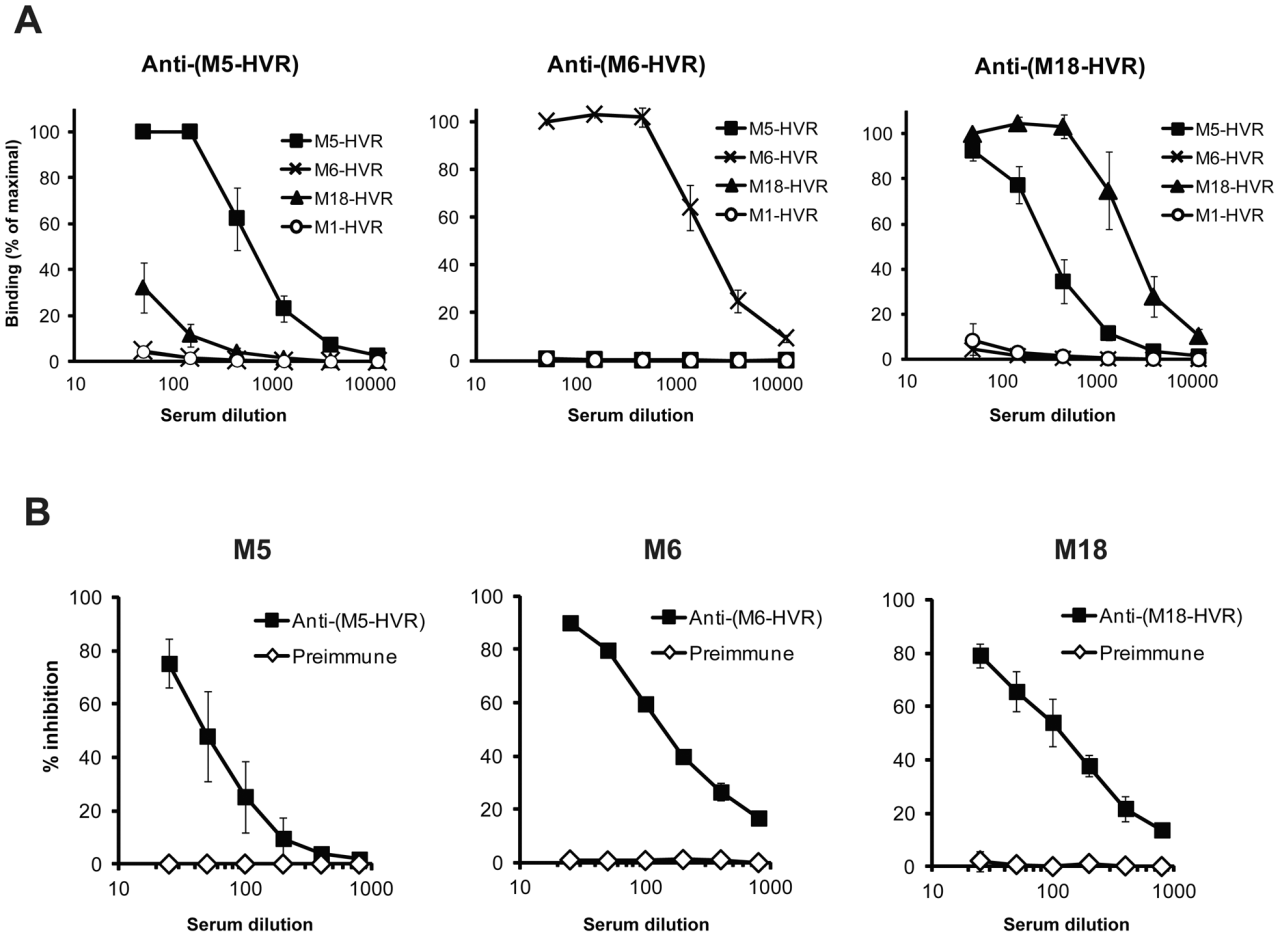
Antigenic properties of FH-binding HVRs. (**A**) Isolated HVRs show limited or no antigenic cross-reactivity. Antiserum to one HVR (specified at the top of a panel) was analyzed for reactivity with four immobilized HVRs, as indicated. The HVRs of M5 and M18 exhibited limited cross-reactivity, while the HVR of M6 did not cross-react with the other two HVRs. The non-FH-binding M1-HVR was included as a control. Pre-immune sera were used to obtain background values, which were subtracted from the values obtained with immune sera. (**B**) Binding of FH to the HVR of an M protein is inhibited by antiserum to the corresponding HVR. The biotinylated M protein indicated above each panel was used to detect FH immobilized in microtiter wells. The binding was inhibited with rabbit anti-HVR serum, diluted as indicated. Preimmune serum was used as a control.

Because the isolated HVRs studied here apparently retain their native structure, as indicated by their ability to bind FH, it was possible to directly compare their antigenic properties. Such comparison was performed with rabbit antisera raised against the isolated HVRs (Figure 5A). In this analysis, the M6-HVR did not cross-react with the other two HVRs, while the HVRs of M5 and M18 exhibited limited cross-reactivity. None of the antisera reacted with the non-FH-binding M1-HVR, used as control. The cross-reactivity between M5 and M18 was not surprising, because these two HVRs exhibit the highest residue identity among the three HVRs studied here (Fig. 3D). Thus, the FH-binding HVRs show little or no antigenic cross-reactivity, although they all bind the same ligand.

To analyze whether antibodies directed against an HVR block FH-binding, the three FH-binding M proteins studied here were mixed with rabbit antiserum to the corresponding HVR and analyzed for ability to bind FH (Figure 5B). In this analysis, binding of FH was efficiently blocked by anti-HVR antibodies but not by preimmune serum, suggesting that anti-HVR antibodies appearing in an infected human may block the binding of FH to an M protein. This result was expected, but not obvious, because polyclonal antibodies may bind to a bacterial protein without blocking its function [49].

### Binding of FH is neither sufficient nor necessary for the ability of an M protein to promote phagocytosis resistance in whole human blood

The combined data available in the M5 system suggest that the FH-binding ability of this M protein does not contribute to phagocytosis resistance, as evaluated in the whole blood assay. This conclusion follows from the finding that the M5 mutant ΔN2 completely lacked ability to bind FH (Figure 2A) but remained resistant to phagocytosis [20]. To further analyze the role of FH-binding in phagocytosis resistance, we studied the M1 and M3 proteins, which did not show detectable binding of FH in our immunochemical analysis (Figure 1C and S1).

**Figure 6:**
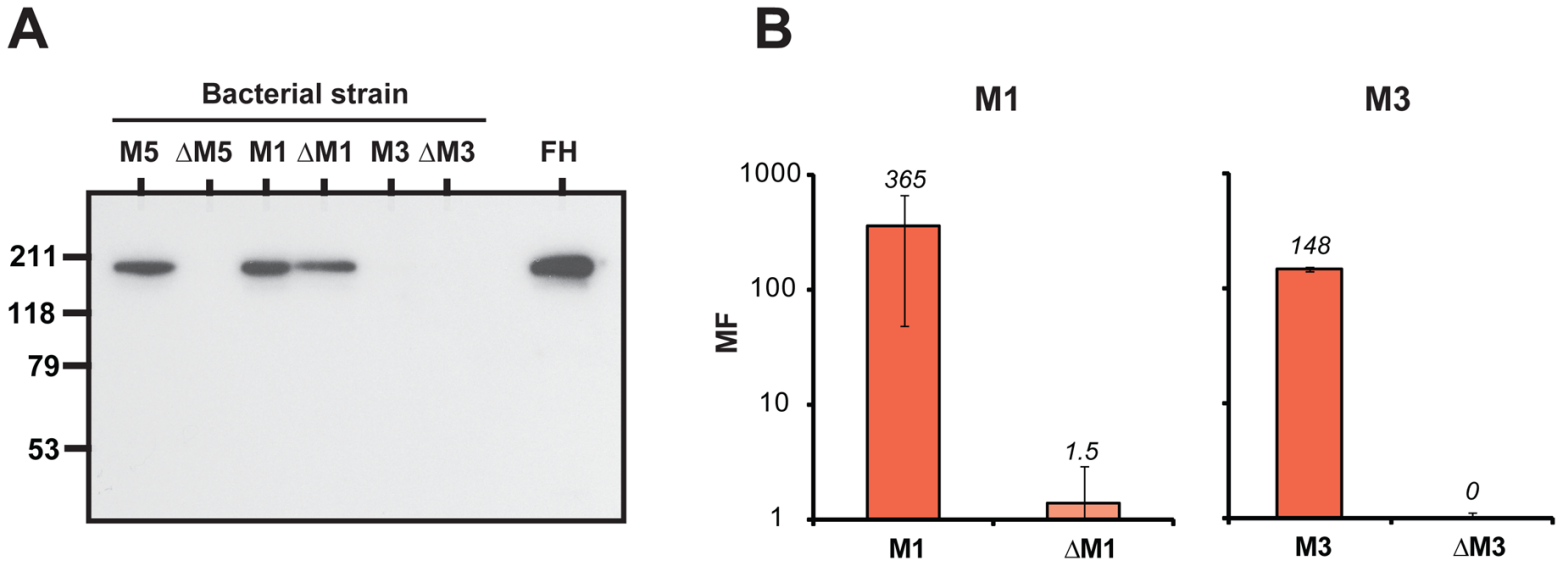
Phagocytosis tests with M1 and M3 strains. (**A**) Suspensions of the bacterial strains indicated were analyzed for ability to bind human FH. After incubation of a bacterial suspension with pure FH (50 µg/ml), the bacteria were washed twice and bound protein was eluted and analyzed by western blot, employing anti-FH for detection. The analysis included wild type M5, M1 and M3 strains and their M-negative mutants (ΔM5, ΔM1 and ΔM3, respectively). Pure FH was included as a control in the blot (right). (**B**) Phagocytosis assay in whole human blood with M1 and M3 strains, and M-negative mutants, as indicated. MF, multiplication factor.

The ability to bind FH was first analyzed for whole bacteria expressing M1 or M3, and isogenic M-negative mutants. As a control, M5-positive and -negative strains were included (Figure 6A). The procedure employed was similar to that used for the analysis of FH-binding strains reported in Figure 1D, i.e. bacteria were incubated with human FH, washed and analyzed for bound protein. In this analysis, the M1-positive and -negative strains were able to bind FH. This result was expected, because M1 strains express the FH-binding Fba protein [50], which is unrelated to M proteins [51]. In contrast, no FH-binding was observed for the M3-positive and -negative strains, reflecting the inability of the M3 protein to bind FH and the absence of Fba from M3 strains [51]. These results do not formally exclude that M3 binds FH with an affinity too low to allow detection under the experimental conditions we used, but this seems unlikely, because the analysis was performed with a relatively high concentration of FH (50 µg/ml) and few washes.

Phagocytosis tests in whole human blood were performed with the M1 and M3 strains (Figure 6B). The results unequivocally showed that the M-positive strains were resistant to phagocytosis, while the M-negative strains were sensitive, in agreement with the classical identification of M proteins as antiphagocytic. Thus, the M1 protein confers phagocytosis resistance although this M protein does not bind FH, and the M1-negative strain is phagocytosis sensitive, although it binds FH. Moreover, the M3 protein conferred phagocytosis resistance although this M protein did not bind detectable amounts of FH. Together, these data indicate that binding of FH to *S. pyogenes* is neither sufficient nor necessary for phagocytosis resistance.

### *In vivo* role of bound FH: analysis with transgenic and wild-type mice

The specificity with which FH binds to the HVR of some M proteins suggested that FH-binding contributes to bacterial virulence, even if bound FH does not contribute to phagocytosis resistance in human blood. In an attempt to prove this hypothesis, we used the Tg mice described above [48], which express a chimeric FH that binds M protein (Figure 4B). If recruitment of FH to an M protein promotes bacterial virulence, one would expect the corresponding *S. pyogenes* strain to be more virulent in Tg mice than in wt mice. The Tg mice were well suited for this analysis, because the serum concentration of chimeric FH is similar to that of FH in wt mice, because the chimeric FH includes the parts of mouse FH implicated in complement regulatory activity, and because the part derived from human FH included the Y402 residue, which may favor binding to M protein [24]. Moreover, the chimeric FH is functional *in vivo*, as determined by ability to prevent C3 consumption [48], [52].

**Figure 7:**
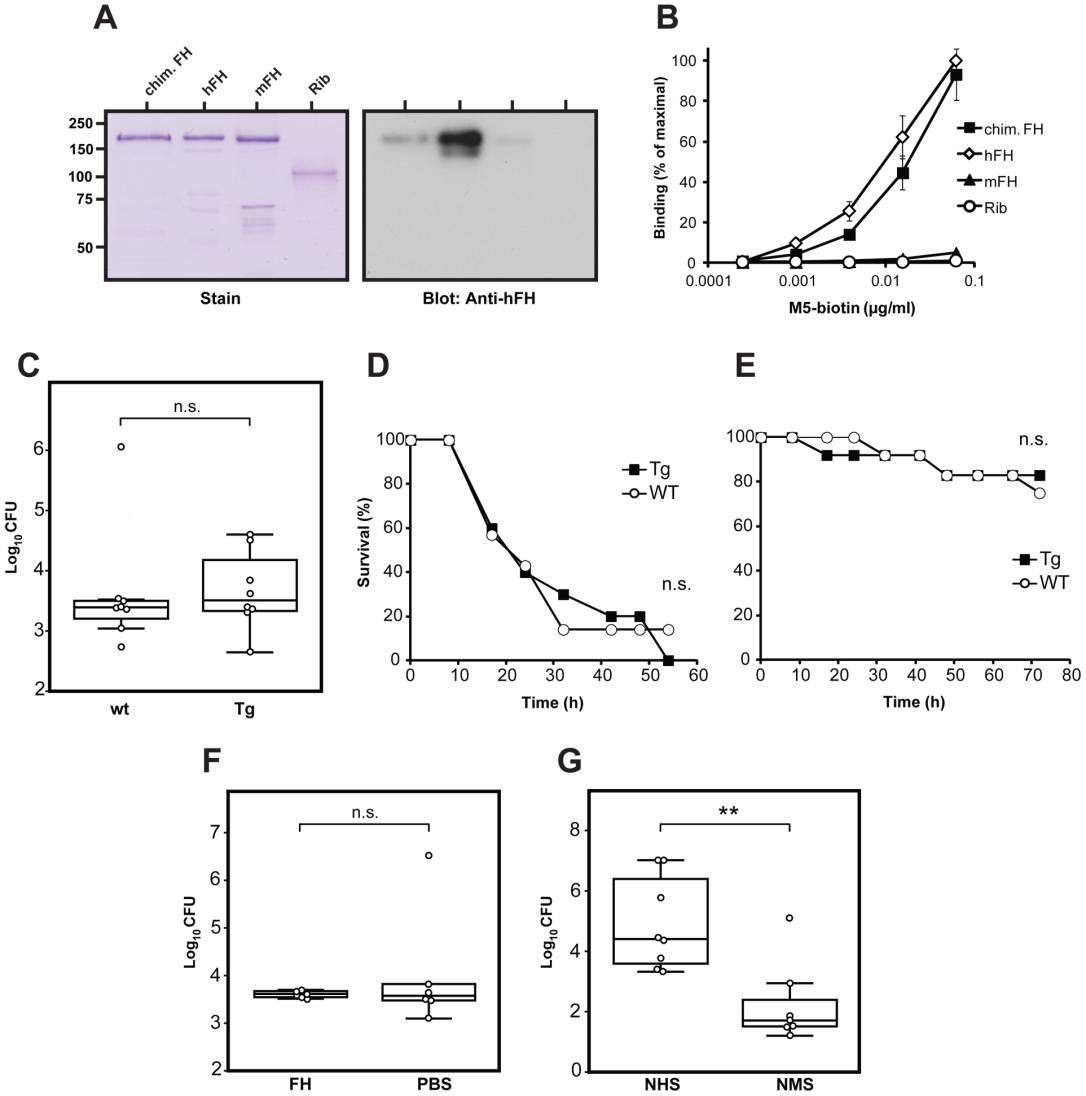
Characterization of a chimeric FH expressed by Tg mice, and infection experiments with Tg and normal mice. (**A**) SDS-PAGE and western blot analysis of purified chimeric FH (chim. FH), derived from mouse and human FH. This FH was compared with pure human Y402 FH (hFH) and pure mouse FH (mFH). Streptococcal protein Rib [81] was included as negative control. The blot was probed with antiserum to human FH. Bound antibodies were detected with radiolabeled protein G followed by autoradiography. (**B**) Binding of the M5 protein to immobilized FH proteins. The proteins used were those shown in panel A. The wells of microtiter plates were coated with the protein indicated, using 0.1 µg protein per well, with protein Rib as negative control. The immobilized proteins were analyzed for ability to bind biotinylated pure M5 protein, added at the concentrations indicated, and bound M protein was detected by the addition of radiolabeled streptavidin. (**C**) Comparison of bacterial growth in wild type mice and Tg mice. The mice (n = 8 for wt and n = 8 for Tg) were subjected to invasive infection with a sublethal dose of the *S. pyogenes* M5 strain and sacrificed after 18 h, followed by determination of bacterial counts in the spleen. (**D**) Survival of wild type (wt) and Tg mice after infection with M5 bacteria. The mice (n = 7 for wt and n = 10 for Tg) were subjected to invasive infection with an ∼LD_90_ dose of the *S. pyogenes* M5 strain and survival was recorded regularly, as indicated. (**E**) Survival of wild type (wt) and Tg mice after infection with M5 bacteria. The mice (n = 12 for wt and n = 12 for Tg) were subjected to invasive infection, as in D, but with a lower bacterial dose. (**F**) Growth of the *S. pyogenes* M5 strain in wild type mice injected with pure human Y402 FH (2×100 µg) or with PBS. The mice (n = 6 for FH and n = 6 for PBS) received FH or PBS 4 h before the infection and mixed with the infecting bacteria. Bacterial counts in spleens were analyzed, as in (C). (**G**) Growth of the *S. pyogenes* M5 strain in wild type mice injected with normal human serum (NHS) or normal mouse serum (NMS). The mice (n = 8 for NHS and n = 7 for NMS) were injected with two 200 µl doses of serum, one dose given 4 h before the infection and one mixed with the infecting bacteria. Analysis as in (C).

For use of the Tg mice in infection experiments, the chimeric FH should have binding properties similar to those of human FH, with regard to M protein. To analyze whether this was the case, we purified the chimeric FH and compared it with pure human FH and also with pure mouse FH (Figures 7A and 7B). Because the chimeric FH was derived from the Y402 allelic variant, pure Y402 human FH was used for the comparison. In SDS-PAGE, the chimeric FH migrated like the human Y402 FH and mouse FH. However, western blot analysis with anti-human FH demonstrated that the three FH proteins had different reactivity, as expected (Figure 7A). While good reactivity was observed for human FH, the chimeric FH reacted weakly, and mouse FH hardly reacted at all under these conditions. When these three pure FH preparations were immobilized in microtiter wells and tested for ability to bind the M5 protein, the chimeric FH and human FH had similar dose-dependent ability to bind M5, while mouse FH did not bind M5 (Figure 7B). These data indicated that the chimeric FH had the desired properties and confirmed the species specificity of FH binding.

Infection experiments with Tg mice were performed with the *S. pyogenes* M5 strain. Mice were subjected to i.p. infection with a sublethal dose and were sacrificed after 18 h, followed by determination of bacterial counts in the spleen. Thus, the experiment analyzed whether the ability to bind FH enhances bacterial virulence during the acute stages of an invasive infection. No difference was seen between the two groups (Figure 7C). In a second type of analysis, the survival of Tg and wt mice was compared after infection with an ∼LD_90_ dose of M5 bacteria. Again, no difference was seen between the two groups, which succumbed to infection with the same kinetics (Figure 7D). Because most of the mice died rapidly in this experiment, it seemed possible that too large a dose of bacteria might have been used, obscuring a difference between the two types of mice. However, similar results were obtained in an experiment in which a lower bacterial dose was used and most of the mice survived (Figure 7E). To analyze whether the route of infection influenced the results, we also infected mice i.n. and determined bacterial counts in lungs after 18 or 42 h. Again, no difference was seen between Tg and wt mice (data not shown). Thus, studies with Tg mice did not provide evidence that FH-binding ability promotes virulence during the acute stages of an infection.

The Tg mouse model appeared to be optimal for analysis of the role of M protein-bound FH in *S. pyogenes* infection. Nevertheless, it seemed possible that intact human FH would work better than chimeric FH in promoting *S. pyogenes* virulence. To analyze this possibility, we used a model in which pure human Y402 FH (total 200 µg) or only PBS was administered i.p. shortly before and at infection with the M5 strain. This model could be used, because human FH can act as a complement regulator in the mouse [53]. In this analysis, there was no difference in spleen colonization between the two groups that received pure FH or PBS (Figure 7F). In contrast, administration of whole human serum (total 400 µl, containing an amount of FH similar to that administered in pure form) strongly increased growth of the M5 strain in spleens, as compared to control mice receiving whole mouse serum (Figure 7G). This result can most simply be explained by the presence in serum of plasminogen, which is known to strongly enhance *S. pyogenes* virulence in a species specific manner [54], but another human serum protein could also have caused the effect. Of note, this analysis indicated that the mouse infection model used here could be employed to detect an enhancement of virulence. In the experiment with pure FH, it is possible that the amount of FH administered was too small, but the results are in agreement with the studies employing Tg mice.

## Discussion

The data reported here provide new information concerning the ability of different M proteins to bind FH, concerning the binding site for FH in an M protein, and concerning the biological role of bound FH. In particular, we found that FH solely binds to the HVR of an M protein and apparently is not needed for phagocytosis resistance or acute virulence. Given these findings, it is of relevance to consider what is known about FH-binding to M protein and its possible biological role.

### M proteins vary in ability to bind FH and C4BP

Our studies of seven purified M proteins demonstrated that different M proteins vary dramatically in ability to bind FH and C4BP, the two major complement regulators in human plasma. Indeed, the data suggest that M proteins may be divided into at least three groups, depending on their ability to bind either FH or C4BP, or neither of these proteins. Thus, ability to bind FH is not a general property of M proteins. Although we cannot exclude that binding of FH with very low affinity escaped detection in the assays employed here, the data demonstrate striking differences in binding ability among different M proteins.

The demonstration that the M5, M6 and M18 proteins bind FH is in agreement with previous studies employing purified proteins and mutant strains [7], [16], [17], [55], and our results suggest that the strains of these M types used here express a single major FH-binding protein, the M protein. For the other two class I proteins studied here, M1 and M3, the situation has remained unclear. While one study employing whole bacteria showed lack of binding to M1 and M3 strains [21], another study indicated that M1 strains bind FH, not via the M1 protein but via the Fba protein, which is unrelated to M proteins [50]. The apparent discrepancy between these two studies may be explained by degradation of FH-binding surface proteins by the SpeB protease, resulting in loss of FH-binding, unless the bacteria are grown in the presence of a SpeB inhibitor [56]. Thus, binding studies performed with whole bacteria must be interpreted with caution. Indeed, we have noted dramatic loss of FH-binding ability if *S. pyogenes* is not grown in the presence of such an inhibitor (data not shown).

Although FH does not bind to all M proteins, the ability to bind this ligand may be an important property of some M proteins, e.g. by favoring one type of infection. Such a situation has been described for the variable *Plasmodium falciparum* PfEmp1 protein, in which the presence of certain regions is associated with specific types of malaria [57], [58]. For the FH-binding M5, M6 and M18 proteins, it is of interest that strains of these M types have been epidemiologically associated with rheumatic fever, the major cause of mortality following *S. pyogenes* infection [13], [29].

### Binding of FH to HVRs

The HVR of an M protein most likely plays a key role in pathogenesis, otherwise it would be eliminated by deletion [59]. Insight into the role of different HVRs is therefore essential for an understanding of *S. pyogenes* infections [26], [59], [60]. Our studies demonstrate that FH binds to the HVR of the M5, M6 and M18 proteins and strongly suggest that these M proteins do not have a second binding site for FH. In particular, several types of analysis indicated that the C repeat region of M5 or M6 does not bind FH. This result is not in agreement with an early immunochemical study suggesting that FH binds to the conserved C repeat region of the M6 protein [16], but fits with our finding that FHL-1, a naturally occurring FH splice variant, binds to the HVR of the M5 protein and probably also the M6 protein [17]. Previously, it appeared possible that FH and FHL-1 bind to different regions of an M protein, but the combined data now strongly indicate that these human proteins solely bind to the HVR of an M protein. The reason for the discrepancy between our results and those reported earlier [16] is unclear, but it is conceivable that the C-terminal M6 fragment used in the early study was contaminated with FH-binding material. Moreover, the peptide inhibition tests reported in that study did not include a control for unspecific inhibition. Concerning binding tests with whole bacteria (Figure 2D and E), the discrepancy between our results and earlier work [19], [38] is less obvious, because at least one of the earlier reports indicated that a surface-expressed M6 protein lacking C repeats retained some ability to bind FH, although binding was lower than for intact M6 [38]. One possible explanation for the different result reported here is that the mutant M6 protein lacking C repeats might show increased sensitivity to the secreted SpeB protease. We avoided this potential problem by growing the bacteria in the presence of an SpeB inhibitor.

Because we only studied a limited number of the ∼200 known M-types, many M proteins probably have an HVR that binds FH. It can be surmised that the FH-binding HVRs of these M proteins will have the same properties as those studied here, exhibiting extensive sequence divergence and little or no antigenic cross-reactivity but identical binding properties. The sequence divergence may have arisen through selection of antigenic escape variants retaining ability to bind FH, an argument implying that bound FH favors bacterial virulence.

Interestingly, the FH-binding HVRs could be studied as isolated polypeptides that retained ability to specifically bind FH. Thus, these HVRs correspond to distinct FH-binding domains, a property that allowed direct immunochemical comparisons and may facilitate future biochemical and structural studies. Similarly, other ligand-binding regions of M proteins can be studied in isolated form, as demonstrated for C4BP-binding HVRs [26], IgA-binding regions [37], and Fg-binding regions [59], [61]. These findings suggest that the fibrillar M protein may be envisaged as a string of domains, representing regions that interact with different human proteins.

### The unclear biological role of FH-binding

The biological role of FH-binding to M protein remains unclear. Indeed, it is not even clear that binding of FH occurs under physiological conditions, because the known FH-binding M proteins all bind Fg, which may sterically interfere with FH-binding [17], [21], [62]. Indeed, ability to bind FH/FHL-1 did not influence complement deposition, when the analysis was performed in human plasma, presumably because the Fg in plasma blocked FH-binding under these conditions [18]. However, immunochemical tests and analysis of complement deposition are of necessity performed under *in vitro* conditions that are of uncertain relevance for the *in vivo* situation. This situation focused interest on the use of Tg mice for *in vivo* analysis, and on analysis of FH-binding in the whole blood phagocytosis system, an *ex vivo* system believed to reflect the human *in vivo* situation.

Concerning the *in vivo* role of FH-binding, our studies with Tg mice did not provide evidence that binding of FH to the M5 protein promotes virulence during the acute stages of an infection. Indeed, the Tg mice employed here were not more sensitive than wt mice to the FH-binding M5 strain, as determined by bacterial growth in spleens and studies of survival. Of note, this result indicates that FH-binding does not promote phagocytosis resistance in mouse blood, otherwise one would have expected more bacterial growth in the spleens of Tg animals, because bacteria must pass through blood to reach the spleen.

Concerning growth in human blood, several lines of evidence now indicate that binding of FH (or FHL-1) to M protein does not provide a general explanation for phagocytosis resistance. Indeed, M proteins such as M1 and M3 do not bind detectable amounts of FH but nevertheless confer resistance to phagocytosis. This result does not exclude that an FH-binding M protein such as M5 might recruit FH to promote phagocytosis resistance, but the studies of M5 strongly indicate that this is not the case. In particular, the chromosomal M5 mutant ΔN2 was resistant to phagocytosis [20], although it lacks detectable ability to bind FH, as shown here. While it cannot be formally excluded that this mutant retained ability to bind FH with an affinity that was too low to allow detection, the simplest explanation for our data is clearly that the ability of M5 to promote phagocytosis resistance is independent of FH-binding. This conclusion raises the question how an M protein promotes resistance to phagocytosis. Interestingly, the available evidence suggests that recruitment of human plasma proteins different from FH may play a key role [33]. Work on class I proteins such as M5 has focused interest on the Fg-binding B repeats [20], [34], [63], [64], and work on the class II protein M22 has focused interest on two adjacent N-terminal regions implicated in the binding of C4BP and IgA [33].

In contrast to our conclusions, two recent papers suggest that FH-binding to M protein indeed promotes phagocytosis resistance in *S. pyogenes* [22], [23]. However, some of the strains employed in those studies were sensitive to phagocytosis, making them unsuitable for analysis of phagocytosis resistance, and none of the studies provided evidence that the strains studied expressed an FH-binding M protein. Indeed, both studies were largely focused on M1 and OF positive strains, in which FH-binding most likely was promoted by the Fba protein, which has limited, if any, effect on phagocytosis resistance [50], [51]. In the first of these papers, the authors made the interesting observation that growth of some *S. pyogenes* strains in human blood was affected by the Y402H polymorphism in FH, suggesting that binding of FH affects resistance to phagocytosis [22], but the interpretation of these data is uncertain, because clear results were only obtained with two strains (of types M1 and st369) in which binding of FH most likely was promoted by the Fba protein, not the antiphagocytic M protein. Thus, this finding does not provide information on the role of FH-binding to M protein. In the second study, the authors reported that phagocytosis of *S. pyogenes* in whole blood was promoted by the addition of an FH fragment assumed to inhibit the interaction between FH and M protein [23]. However, the concentration of inhibitor used was much below the level that would be required to cause inhibition of FH-binding in blood, according to other data reported in the same paper, and it was not excluded that the FH fragment had unspecific effects. Thus, neither of the two studies provided conclusive evidence that FH-binding to M protein promotes phagocytosis resistance.

Although FH-binding ability did not promote phagocytosis resistance or virulence in the systems studied here, the specificity of the binding suggests that FH bound to an HVR affects virulence under certain conditions. What could be the function of M protein-bound FH, if it does not promote resistance to phagocytosis? In one possible scenario, FH promotes virulence by promoting adhesion of pathogens to host cells, not by down-regulating complement [65], [66]. For *S. pyogenes* our studies with transgenic mice did not support this hypothesis, because Tg mice were not more sensitive to infection, even when the bacteria were administered via the i.n. route. Moreover, *in vitro* adhesion tests with epithelial cells did not provide evidence that FH promotes *S. pyogenes* adhesion in an M-protein-dependent fashion (data not shown). Possibly, FH bound to M protein does not contribute to virulence during the early stages of an *S. pyogenes* infection but has its major effect later, when down-regulation of complement activation might modulate inflammatory and adaptive immune responses [2], [67], [68]. Thus, bound FH might contribute to *S. pyogenes* virulence by mechanisms not assessed in currently used model systems. This hypothesis is fully compatible with the suggestion that the Y402H polymorphism in FH may affect sensitivity to *S. pyogenes* [23], [24].

### Ligand-binding properties of the HVR in different M proteins

**Figure 8:**
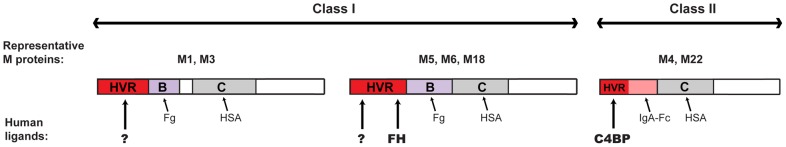
Binding of human proteins to M proteins, with focus on the HVR. The figure summarizes current knowledge in the field, with emphasis on the M proteins studied here. Fg, fibrinogen; HSA, human serum albumin; IgA-Fc, Fc-part of IgA. See text for details.

Because the HVR of an M protein plays a key role in virulence and is a target for protective antibodies, it is of interest to consider what is now known about the ligand-binding properties of different HVRs. Current knowledge is summarized in Figure 8, with focus on the M proteins studied here.

For the M1 and M3 proteins little is yet known about the HVR, but the HVR of M1 was suggested to bind the antibacterial peptide LL-37 [60], [69], and the HVR of M3 was reported to bind a collagen fragment [70]. The HVR of the M5, M6 and M18 proteins binds FH, as reported here. Of note, our data on M5 show that only the C-terminal part of the HVR is absolutely required for FH-binding. It is possible that the N-terminal part of this HVR enhances the affinity and/or the specificity of FH-binding, but it is also conceivable that the HVR has a second function, in addition to FH-binding. Indeed, deletions in the M5-HVR block mouse virulence, although this HVR does not bind mouse FH, suggesting that the HVR makes an FH-independent contribution to virulence [59]. Finally, the HVRs of M4 and M22 bind C4BP [27], and IgA binds to an adjacent region that also is very variable [31], [71]. These two ligand-binding regions in the N-terminal part of M4 and M22 have a combined length that is shorter than the total length of the HVR in the other M proteins considered here, supporting the notion that the HVR of an M protein may have more than one function.

### Concluding remarks

Determination of the *in vivo* relevance of *in vitro* findings represents one of the major challenges in studies of microbial pathogenesis [72], [73]. Our studies of FH-binding and M proteins underline the difficulty in making such extrapolations. Indeed, our data show that the *in vivo* role of FH-binding remains unclear, although it has been taken for granted that this interaction promotes phagocytosis resistance and acute virulence in *S. pyogenes*. This conclusion is particularly surprising, because the binding of a human complement regulator, FH or C4BP, emerges as a property shared by many HVRs, suggesting that these interactions enhance virulence (Figure 8). Thus, our data provide intriguing new information concerning the HVR in M proteins, while suggesting that new experimental systems may be needed to identify the biological role of bound FH.

## Materials and Methods

### Bacterial strains and media

The M1 strain *S. pyogenes* SF370 [74] and its isogenic Δ*emm1* mutant [75], referred to here as ΔM1, were from M. A. Kehoe. The *S. pyogenes* M3 strain 950771 and its isogenic Δ*emm3* mutant 296, referred to here as ΔM3, were from M. Wessels [76]. *S. pyogenes* strain M5 Manfredo [77] was from M. A. Kehoe. The isogenic mutant strains ΔM5, ΔN1, ΔN2, ΔB, and ΔC have been described [17], [20]. *S. pyogenes* JRS4 (M6), and its isogenic mutant strains JRS145 (ΔM6) and JRS251 (M6ΔC) were from J. R. Scott [19]. *S. pyogenes* 87-282 (M18) and its isogenic Δ*emm18* strain 282 KZ (referred to here as ΔM18) were from M. Wessels [78]. All *S. pyogenes* strains were grown without shaking in Todd-Hewitt broth supplemented with 0.2% yeast extract (THY), in 5% CO_2_ at 37°C. Unless otherwise stated, the *S. pyogenes* cultures were cultivated overnight in medium supplemented with the SpeB inhibitor E64 (Sigma), used at 10 µM, to avoid degradation of M protein by the secreted SpeB protease that may be present in stationary phase cultures [56]. *Escherichia coli* XL1 Blue and DH5α were used for cloning and strain BL21 for protein production. *E. coli* was grown in LB at 37°C with shaking and supplemented with 100 µg/ml ampicillin when appropriate.

### Human and mouse proteins

Human FH was from Complement Technology, Inc. This FH, which contains both the Y402 and H402 variants, was used in all experiments, unless otherwise stated. Pure human Y402 FH was purified by affinity chromatography from human serum containing only Y402 FH, using an immobilized construct derived from M proteins (manuscript in preparation). Pure chimeric FH was similarly isolated by affinity chromatography of serum from Tg mice expressing the Y402 variant. Mouse FH was affinity purified from EDTA-plasma on a HiTrap column (GE Healthcare) containing the anti-mouse FH mAb 2A5; protein was eluted with glycine-HCl pH 2.5 and immediately neutralized and dialyzed (C. Harris, in preparation). Purification of human C4BP was described in [41]. Human Fg was from Enzyme Research Laboratories.

### Pure recombinant M proteins, HVRs and mutant M proteins

All recombinant M proteins and M protein fragments, except M4 and M22, were produced as GST-tagged proteins and purified on GSTrap columns according to the manufacturer's instructions (GE Healthcare). After removal of the GST moiety, these recombinant proteins included the N-terminal sequence GPLGS, not present in the original protein. For preparation of the GST-tagged proteins, PCR products were cloned into BamHI-EcoRI cleaved pGEX-6P-2 (GE Healthcare). The genes and gene fragments were amplified from *S. pyogenes* chromosomal DNA, employing the strains described under Bacterial strains and media, using primers listed in Table S1 as follows: M1-HVR (M1-F/M1HVR-dim-R), M3 (M3-F/M3-R), M5-HVR (M5-F/M5HVR-dim-R), M5 (M5-F/M5-R), M6-HVR (M6-F/M6HVR-dim-R), M6 (M6-F/M6-R), M6-Crep (M6C-F/M6C-dim-R), M18-HVR (M18-F/M18-HVR-dim-R) and M18 (M18-F/M18-R). The Pwo DNA polymerase was used for all PCR reactions according to the manufacturer's instructions (Roche). The sequence was confirmed for all cloned PCR fragments. Purification of recombinant M1 protein was described in [79], and the recombinant M4 and M22 proteins were described in [80].

For the preparation of an M5 mutant protein with a deletion corresponding to amino acid residues 80-86 (M5Δ80-86), two PCR fragments were first generated, one with the primer pair M5-F and M5Δ80-86REV, the other with the pair M5Δ80-86FWD and M5-R. A longer PCR fragment encoding M5Δ80-86 was generated by overlap extension PCR, using the two first PCR fragments and primers M5-F and M5-R. This fragment was cloned into pGEX-6P-2. The deletion protein M5Δ87-93 was prepared by a similar procedure, employing the primer pair M5-F and M5Δ87-93REV, and the pair M5Δ87-93FWD and M5-R, followed by overlap extension PCR using primers M5-F and M5-R. This procedure was also followed for the generation of a PCR product encoding a deletion variant of M6 lacking amino acid residues 97-110 (M6Δ97-110). In this case, the two first PCR fragments were generated with primer pairs M6-F/M6Δ97-110REV and M6Δ97-110FWD/M6-R, respectively, and overlap extension PCR was performed with primers M6-F and M6-R.

The recombinant HVRs derived from the M1, M5, M6 and M18 proteins contain the first 91, 121, 129, and 108 amino acids, respectively, of the corresponding mature M proteins, while the M6-Crep construct comprises residues 228-363 of the mature M6 protein. To allow covalent dimerization of these purified M protein fragments, the recombinant forms contained a C-terminal cysteine not present in the intact M protein. For this purpose, a cysteine codon was added in the corresponding DNA constructs. After removal of the GST tag, the HVRs were dimerized as described [26].

### Antisera

Antisera against the dimerized M5, M6 and M18 HVRs were raised by subcutaneous immunisation of rabbits with 100 µg pure protein in complete Freund's adjuvant, followed by two 50 µg boosters in incomplete Freund's adjuvant four and eight weeks after the first immunisation. The rabbits were bled two weeks after the final booster. A similar procedure was used to raise antiserum against highly purified human C4BP [41]. Sheep anti-human FH IgG (The Binding Site) was used to detect human FH, and rabbit anti-human Fg (Dako, Denmark) was used to detect Fg. Wt mouse FH and chimeric FH expressed by transgenic mice was detected with a mouse anti-mouse FH monoclonal antibody (designated 2A5) targeting mouse SCR1-4 (C. Harris, in preparation). Bound mouse Ig was detected with secondary rabbit anti-mouse Ig (Dako, Denmark).

### Binding and inhibition tests with immobilized pure ligands

#### Direct binding tests with immobilized pure proteins and pure added ligands (Figure 1C, 2B, 2C, 3B, 7B, S1, S2B)

Microtiter wells were coated overnight at 4°C, using 50 µl per well of a solution of pure protein in PBS, as indicated. All subsequent steps were performed at RT. After the coating, the wells were blocked for 1 h with TBST-gel (Tris-buffered saline supplemented with 0.25% Tween 20 and 0.25% gelatin). After addition of pure protein ligand at the indicated concentration (in 50 µl of TBST-gel), the wells were incubated for 1 h. Unless otherwise stated, bound ligands were detected by addition of rabbit or sheep antibodies (diluted 500-fold in TBST-gel) targeting the respective ligand and incubation for 1 h. Bound antibodies were detected through addition of radiolabeled protein G (∼10,000 cpm), incubation for 1 h, and determination of bound protein G in a γ-counter. The wells were washed with PBST (PBS with 0.25% Tween 20) between each step. Binding was calculated in percent of the maximal protein G binding. As a control, all experiments included wells coated and treated as the other wells, except that soluble ligand was not added. No significant binding was seen in any of the controls. Thus, the Ig-Fc-binding ability of some M proteins, such as M4( = Arp4) and M22( = Sir22) [32], did not contribute to binding under these conditions.

#### Direct binding tests with immobilized M proteins and mouse sera (Figure 4B)

The analysis was performed essentially as described above. Wells were coated with the M protein indicated, using 0.1 µg protein per well. After blocking, serum from wt or Tg mice was added, diluted as indicated in TBST-gel. Bound mouse FH or chimeric FH was detected through an initial incubation with the mouse anti-mouse FH mAb 2A5 (diluted to 7 µg/ml in TBST-gel), followed by incubations with rabbit anti-mouse Ig (diluted 1000-fold in TBST-gel) and radiolabeled protein G and determination of bound protein G, as described above. Control wells were treated in the same way, except that no mouse serum was added. Of note, the M5, M6 and M18 proteins used in this analysis are not known to have IgG-Fc-binding ability, excluding such activity as a source of background binding. Indeed, no binding was observed in the controls.

#### Inhibition assays with free HVRs and with anti-HVR antibodies (Figure 4C and 5B)

For the analysis with free HVRs (Figure 4C), the conditions were optimized to increase the sensitivity of the test. Pure FH (60 ng) was immobilized in microtiter wells, which were blocked with TBST-gel. Biotinylated M protein (total 1.5–6.5 ng/well, depending on the M protein) was added and the binding was inhibited by mixing of the M protein with pure HVRs, as indicated. For each M protein, the four HVRs tested were added at the same concentration, 10 µM. The M proteins and HVRs were diluted in TBST-gel, but the sensitivity of the assay was increased by 3-fold dilution of all solutions, including washing buffers, with distilled water. Bound M protein was detected using radiolabeled streptavidin (∼10,000 cpm) and determination of bound radioactivity. Binding was calculated in percent of streptavidin bound without inhibitor and this value was subtracted from 100, giving percent inhibition.

For the inhibition analysis with antisera (Figure 5B), biotinylated M protein (total 3 ng/well) was used to detect immobilized FH (0.1 µg/well) and binding was inhibited by the simultaneous addition of heat-inactivated rabbit anti-HVR serum, diluted as indicated. Preimmune serum was used as a control. Binding and inhibition were determined as described above.

#### Analysis of cross-reactivity (Figure 5A)

Wells were coated with 0.1 µg of the FH-binding HVRs indicated, diluted in PBS. After blocking with TBST-gel, the immobilized HVRs were analyzed for reactivity with different rabbit anti-HVR sera, diluted as indicated. Bound rabbit antibodies were detected with radiolabeled protein G. The non-FH-binding M1-HVR was used as control. Pre-immune rabbit sera were used to obtain background values, which were very low and were subtracted from the values obtained with immune sera.

### Binding and elution assays with whole bacteria

The *S. pyogenes* bacteria were harvested from overnight cultures, washed twice with TBS-T (50 mM Tris, 0.15 M NaCl, 0.05% Tween-20, pH = 7.4), and resuspended in the same buffer.

#### Direct binding assays (Figure 2D and 2E)

Samples of 10^8^ cfu were incubated with the indicated amount of FH for 1 h in a volume of 100 µl. After one wash with 2 ml buffer and an additional centrifugation, bound FH was quantified by the addition of sheep anti-human FH (diluted 250-fold in TBST), followed by one wash and detection of bound anti-FH with radiolabeled protein G.

#### Elution assays (Figure 1D, 2A and 6A)

A bacterial sample (1.75×10^9^ cfu) was pelleted, and the bacteria were resuspended in PBS (500 µl) containing 50 µg/ml pure FH, followed by incubation on a shaker for 1 h at RT. After two washes with 5 ml PBS, bound FH was eluted by incubating the resuspended bacteria in 0.2 M glycine pH 2.0 (2.5 ml) for 15 min at RT. Following centrifugation, the supernatant was collected and the pH was adjusted by the addition of 1 M Tris-HCl pH 8.0 (1 ml). The sample was concentrated 10-fold and analyzed for the presence of FH by western blot, using anti-human FH for detection.

### Affinity chromatography of serum on columns with immobilized HVRs

For each of the dimerized HVRs derived from M5, M6 and M18, 600 µg was coupled to a 1 ml HiTrap NHS-activated HP column according to the manufacturer's instructions (GE Healthcare). For the generation of a control column, reactive groups were inactivated with ethanolamine. Outdated human citrate plasma, purchased from Lund University Hospital Blood Centre, was converted to serum by dialysis against 50 mM Tris-HCl pH 7.2, 137 mM NaCl, 2.7 mM KCl, 5 mM CaCl_2_ at 4°C. The clot was removed and the serum was frozen until use. Mouse (C3H/HeN) and rabbit sera were obtained after coagulation of freshly drawn blood. Prior to use, frozen sera were thawed and particulate matter was removed by filtration (0.45 µm).

Column chromatography was performed at 4°C. The columns were initially equilibrated with 10 column volumes of PBS. The various sera (1.5 ml for Figure 3C and 0.5 ml for Figure 4A) were diluted 3 times in PBS and applied with a flow rate of 0.055 ml/min, followed by washes with 10 column volumes of PBS at a flow rate of 1 ml/min. Protein was eluted in 5 ml 6 M guanidine-HCl, dialysed against PBS and concentrated 10-fold.

### Phagocytosis assays

The assays were performed essentially as described [33], using hirudin as anticoagulant with freshly drawn human blood from “nonimmune” donors, i.e. blood allowing rapid growth of the M-positive strains studied. The assay employed a very small inoculum of log-phase bacteria, grown in medium without E64. After rotation at 37°C for 3 h, the multiplication factor was calculated for each strain. Assays were performed with blood from three different donors (M1) or two donors (M3).

### Mouse infection models

The transgenic mice used (on the C57Bl/6 background, bred as hemizygotes) express a chimeric human/mouse FH in which SCRs 6–8 are derived from human FH, with a tyrosine residue at position 402 [48]. Infection experiments were performed with the *S. pyogenes* M5 Manfredo strain, using log-phase bacteria grown in medium without E64. For analysis of the effect of the chimeric FH on spleen colonization (Figure 7C), male Tg and wt male litter mates were challenged i.p. with a sublethal dose of bacteria (2×10^6^ cfu). Mice were sacrificed 18 h post challenge, when spleens were homogenized and analyzed for the presence of bacteria by standard pour-plate methods. For analysis of the effect of the chimeric FH on lethal infection, two studies were performed. In one study (Figure 7D), female Tg and wt female litter mates were challenged i.p. with an ∼LD_90_ dose (2.0×10^7^ cfu) of M5 bacteria and survival was followed. In the second study (Figure 7E), the mice used were male and received a lower dose of M5 bacteria (0.9×10^7^ cfu).

For studies with non-Tg mice (Figures 7F and G), pure human Y402 FH or whole human serum was administered i.p. to C3H/HeN mice 4 h before and also simultaneously with the i.p. administration of a sublethal dose of bacteria (2×10^7^ cfu for mice of this inbred strain). Spleens harvested after 18 h were analyzed for the presence of bacteria. In the experiment with pure FH, the mice (female) received human Y402 FH (2×100 µg) or PBS; the FH administered together with the bacteria was preincubated with the bacteria for 30 min at RT before challenge. In the experiment with human serum, the mice (male) received 200 µl at the two time points. Control mice received mouse serum.

### Other methods

Protein G and streptavidin were purchased from Sigma and radiolabeled as described [81]. Biotinylation of pure recombinant M5, M6 and M18 proteins was performed using the EZ-Link Sulfo-NHS-LC-Biotinylation kit according to the manufacturer's instructions (Pierce). Western blot and detection of bound antibodies with radiolabeled protein G was performed as described [81]. Mass spectrometric identification of purified proteins was performed by the SCIBLU Proteomics Resource Centre at Lund University (details available on request). N-terminal sequencing of proteins was performed by Alphalyse, Denmark. Protein sequence alignments were performed using the ClustalW program (http://www.ebi.ac.uk/Tools/msa/clustalw2/).

### Statistical analysis

Results from binding and phagocytosis assays are presented as mean values with SD from three independent determinations. In Figure 7, the horizontal lines within the boxes represent the median. The boxes represent the interquartile ranges, IQR, and the t-bars the lowest normal datum still within 1.5 IQR of the lower quartile, and the highest normal datum still within 1.5 IQR of the upper quartile. To compare cfu numbers between groups, the Mann-Whitney U test was used. The statistical analyses were performed in SPSS Statistics 18 for Windows (IBM Corporation, Somers, NY, USA). Significance (p<0.01) is indicated by **.

### Ethics statement

Studies with human serum employed outdated human citrate plasma, converted to serum as described above. The plasma samples, which were anonymized, were purchased from Lund University Hospital Blood Centre, with permission (2012:04). Phagocytosis tests were performed with blood samples obtained from human volunteers, with permission from the Ethical Review Board of the Medical Faculty, Lund University (2012/290) and with written informed consent from the donors. Animal experiments were performed with permission from the Animal Experimental Ethics Committee at Lund District Court (M23-08; M286-09; M284-09; M129-11; M34-12). Experimental infections were performed in a level P2 biohazard laboratory within the animal facility of Department of Laboratory Medicine, Lund University, and were governed by the following directive, law and provisions: Council directive EG 86/609/EEC, the Swedish Animal Welfare Act (1988:534) and the Swedish Animal Welfare Ordinance (1988:539). Provisions regarding the use of animals for scientific purposes: DFS 2004:15, DFS 2005:4, SJVFS 2001:91, SJVFS 1991:11.

## Supporting Information

Figure S1 Dose-dependent binding of pure human FH to pure M proteins immobilized in microtiter wells. The wells were coated with 0.1 µg M protein and increasing amounts of FH were added, using 50 µl FH solution of the concentration indicated. Bound FH was detected by incubation with specific antibodies, followed by radiolabeled protein G.
(TIF)

Figure S2 Characterization of M5 and M6 derivatives employed to study the binding site for FH. (**A**) SDS-PAGE of purified M5 and M6 deletion derivatives with short truncations in the C-terminal part of the HVR. See Figure 2B and 2C for the location of the truncations. (**B**) Analysis of fibrinogen (Fg)-binding ability of the five proteins shown in panel (A). The M proteins were immobilized in microtiter wells, using 0.1 µg protein per well, and the immobilized protein was analyzed for ability to bind added Fg, as indicated. (**C**) SDS-PAGE of the M6-Crep construct, derived from the C repeat region of the M6 protein (see Figure 2C). The intact M6 protein was included for comparison. The M6-Crep construct was dimerized via a C-terminal Cys residue not present in the native M6 protein, which does not contain any Cys residues. The electrophoresis was run under reducing and non-reducing conditions, as indicated.
(TIF)

Table S1 Primers used for PCR amplification. The underlined nucleotide sequences hybridize to the target genes. Endonuclease cleavage sites are indicated in bold. BamHI and EcoRI were used to insert fragments into the pGEX-6P-2 vector. The forward-primers designated M1-F etc. were used to amplify both full-length constructs and the corresponding HVR constructs. The reverse primers used to amplify HVR constructs introduced a C-terminal cysteine residue, used for dimerization, and are labeled “dim”.
(DOC)
