# Supplementary figures and images for: Factor H Binds to the Hypervariable Region of Many Streptococcus pyogenes M Proteins but Does Not Promote Phagocytosis Resistance or Acute Virulence
Source: PLoS Pathog. 2013 Apr 18;9(4):e1003323. doi: 10.1371/journal.ppat.1003323 (PMC3630203; doi:10.1371/journal.ppat.1003323)

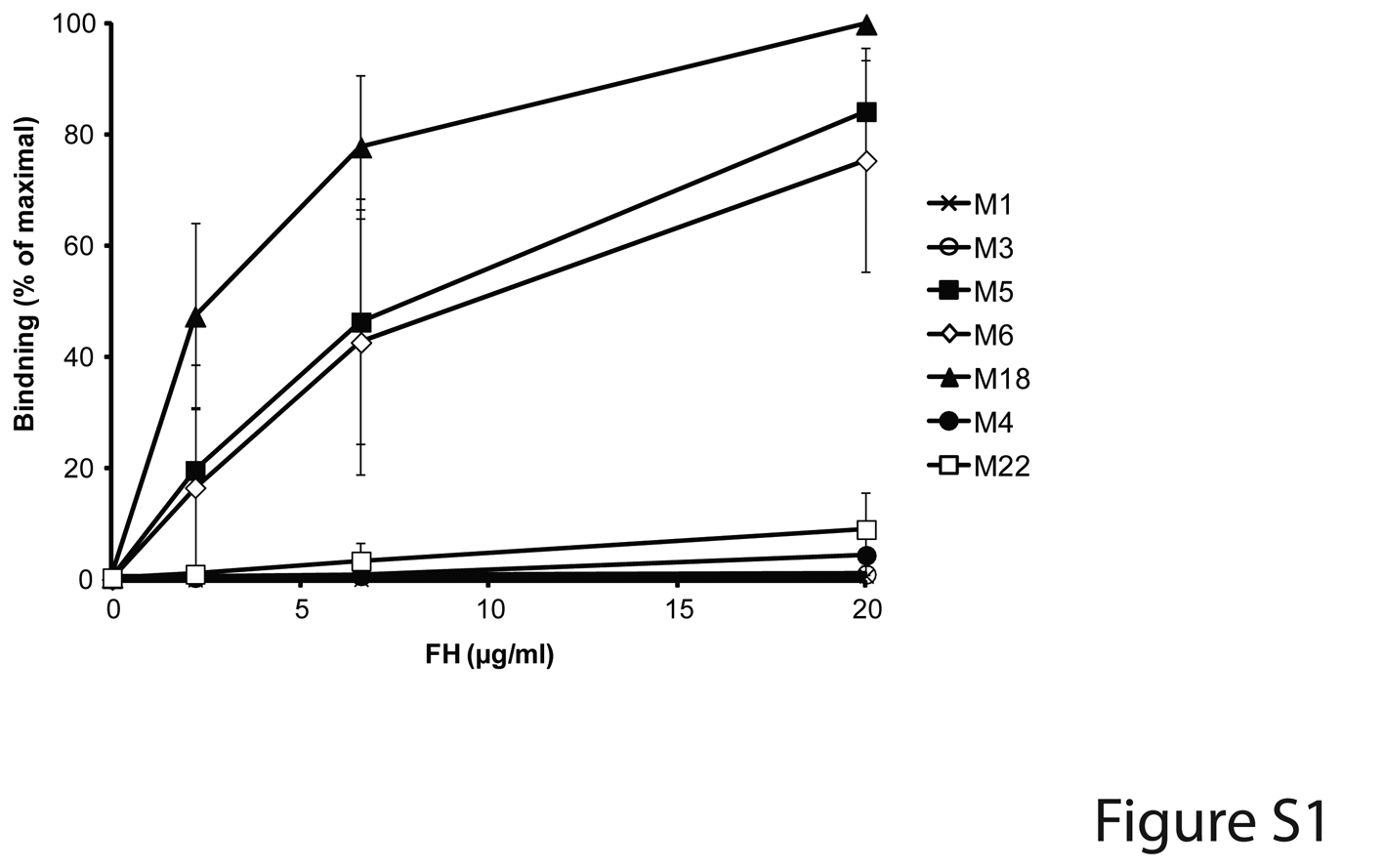

Supplement: Figure S1 — Dose-dependent binding of pure human FH to pure M proteins immobilized in microtiter wells. The wells were coated with 0.1 µg M protein and increasing amounts of FH were added, using 50 µl FH solution of the concentration indicated. Bound FH was detected by incubation with specific antibodies, followed by radiolabeled protein G. (TIF) [file ppat.1003323.s001.tif]

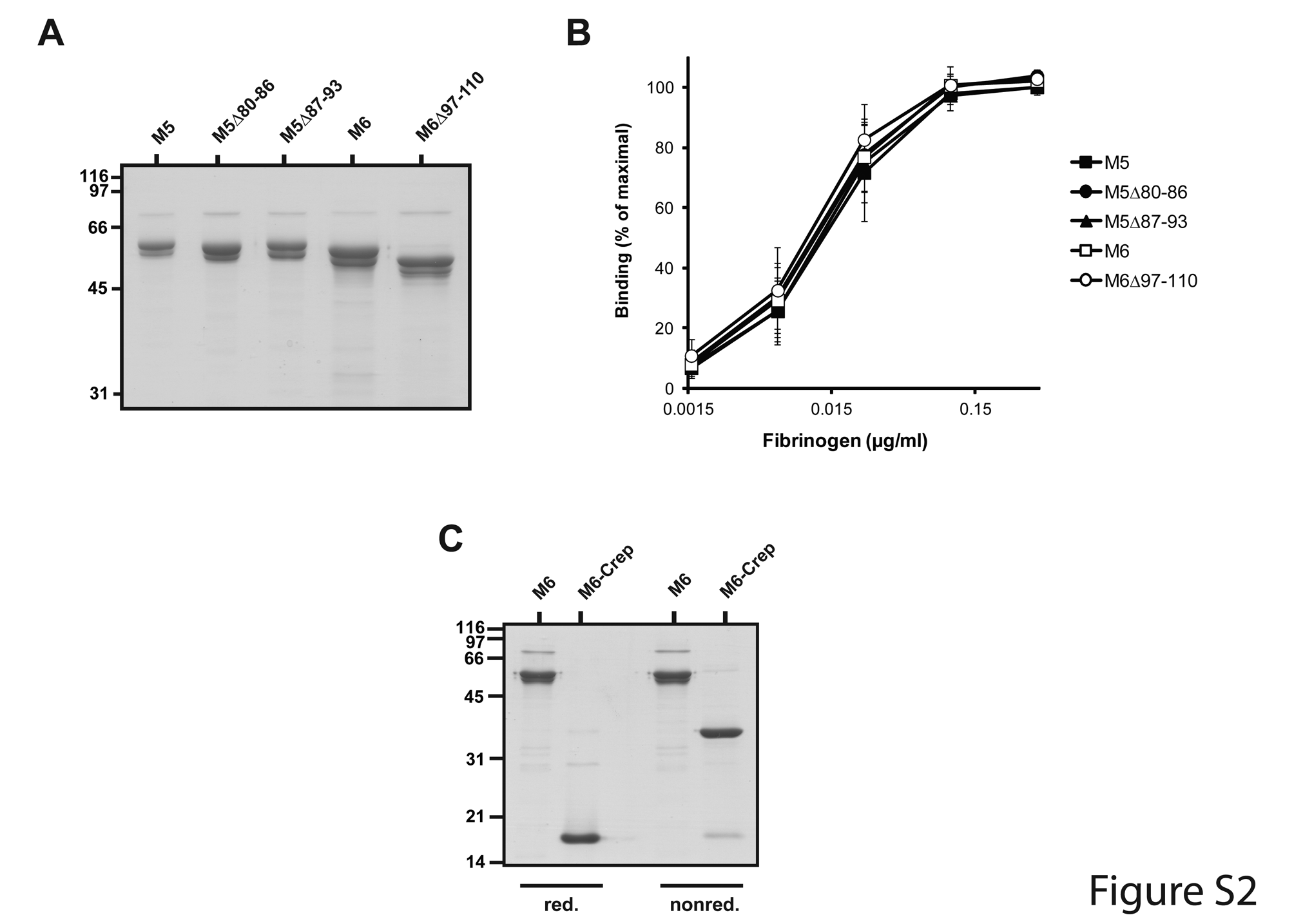

Supplement: Figure S2 — Characterization of M5 and M6 derivatives employed to study the binding site for FH. (A) SDS-PAGE of purified M5 and M6 deletion derivatives with short truncations in the C-terminal part of the HVR. See Figure 2B and 2C for the location of the truncations. (B) Analysis of fibrinogen (Fg)-binding ability of the five proteins shown in panel (A). The M proteins were immobilized in microtiter wells, using 0.1 µg protein per well, and the immobilized protein was analyzed for ability to bind added Fg, as indicated. (C) SDS-PAGE of the M6-Crep construct, derived from the C repeat region of the M6 protein (see Figure 2C). The intact M6 protein was included for comparison. The M6-Crep construct was dimerized via a C-terminal Cys residue not present in the native M6 protein, which does not contain any Cys residues. The electrophoresis was run under reducing and non-reducing conditions, as indicated. (TIF) [file ppat.1003323.s002.tif]
