# Supplementary material for: Factor H Binds to the Hypervariable Region of Many Streptococcus pyogenes M Proteins but Does Not Promote Phagocytosis Resistance or Acute Virulence
Source: PLoS Pathog. 2013 Apr 18;9(4):e1003323. doi: 10.1371/journal.ppat.1003323 (PMC3630203; doi:10.1371/journal.ppat.1003323)
Supplement: Table S1 — Primers used for PCR amplification. The underlined nucleotide sequences hybridize to the target genes. Endonuclease cleavage sites are indicated in bold. BamHI and EcoRI were used to insert fragments into the pGEX-6P-2 vector. The forward-primers designated M1-F etc. were used to amplify both full-length constructs and the corresponding HVR constructs. The reverse primers used to amplify HVR constructs introduced a C-terminal cysteine residue, used for dimerization, and are labeled “dim”. (DOC) [file ppat.1003323.s003.doc]

**Table S1. Primers used for PCR amplification**

# M1-F: ATATGGATCCAACGGTGATGGTAATCCTAGG

# M1HVR-dim-R: GCATGAATTCTTATTAGCATCTTTGTCTATCCCAAC

# M1-R ATATGAATTCTTATTAGTTTTCTTCTTTGCGTTTTACA

# M3-F: ATATGGATCCGATGCTAGGAGTGTTAATGGAGAGT

# M3-R: Identical to M1-R

# M5-F: ATATGGATCCGCCGTGACTAGGGGTAC

# M5HVR-dim-R: ATATGAATTCTTATTAGCACTCTTGCTGTTTATTTGCTA

# M5-R: ATATGAATTCTTATTAATTTTCTTCTTTGCGTTTTACAA

# M6-F: GCATGGATCCAGAGTGTTTCCTAGGGGGAC

# M6HVR-dim-R: GCATGAATTCTTATTAGCACTCATCCAAGGTTTTTTTAAGG

# M6-R: Identical to M1-R

# M6C-F: ATATGGATCCGCTAAAAAAGATGAAGGAAACA

# M6C-dim-R: ATATGAATTCTTATTAGCATCCAGCTCTTAGTTTTGCA

# M18-F: ATATGGATCCGCACCTCTTACTCGAGCTA

# M18HVR-dim-R: Identical to M5HVR-dim-R

# M18-R: Identical to M1-R

# M5Δ80-86FWD GATACGTTATCTACTCAGGTACAGAACACGCAATAC

# M5Δ80-86REV GTATTGCGTGTTCTGTACCTGAGTAGATAACGTATC

# M5Δ87-93FWD GAAACTCTTGAAAGAGAAAATGAAACGTTAAAGATTAAG

# M5Δ87-93REV CTTAATCTTTAACGTTTCATTTTCTCTTTCAAGAGTTTC

# M6Δ97-110FWD TAGGTTAACAACTGAGAATAAAAATAAAGAGCGAGAAAATAAAG

# M6Δ97-110REV CTTTATTTTCTCGCTCTTTATTTTTATTCTCAGTTGTTAACCTA
